# Measuring Spatial Abilities in Children: A Comparison of Mental-Rotation and Perspective-Taking Tasks

**DOI:** 10.3390/jintelligence11080165

**Published:** 2023-08-16

**Authors:** Andrea Frick, Stefan Pichelmann

**Affiliations:** Department of Psychology, University of Fribourg, 1700 Fribourg, Switzerland; stefan.pichelmann@unifr.ch

**Keywords:** children, spatial cognition, assessment, perspective taking, mental rotation

## Abstract

Mental rotation (MR) and perspective taking (PT) are important spatial abilities and predictive of performance in other cognitive domains. Yet, age-appropriate measures to assess these spatial abilities in children are still rare. This study examined psychometric properties of four MR tasks in 6- to 9-year-olds (*N* = 96). Two were developed specifically for children and two were based on established assessments for adults; one of each was a computerized and one was a paper–pencil task. Furthermore, spatial perspective taking (PT)—a different but closely related ability—was assessed to determine discriminant validity. Factor analyses showed that all MR tasks loaded on one single factor, with PT only loading weakly on the same factor, suggesting high construct validity. The computerized task for adults showed moderate factor loadings, constituted its own (but correlated) factor when a two-factor solution was forced, and showed the lowest reliabilities, suggesting that it was very difficult for children. On average, the new MR tasks had good to excellent reliabilities, differentiated well between age groups, and proved to be well-suited to assess MR in this age range. The PT task also showed good reliability and a steep developmental progression. Relations to verbal skills, gaming experience, and TV consumption are discussed.

## 1. Introduction

### 1.1. Individual Differences in Spatial Abilities

Over the past century, research on cognitive processing and intelligence has highlighted the importance of spatial reasoning (cf. Atit et al. 2020), and spatial intelligence or ‘visualization’ has been proposed as a major component in many theories of intelligence (e.g., Gardner 1983; Carroll 1993). In the current spatial literature, the prevailing view is that “spatial ability” does not refer to a homogeneous construct but should be understood as a collective term for a wide variety of abilities (Newcombe and Shipley 2015; Uttal et al. 2013; Linn and Petersen 1985; McGee 1979) that typically vary greatly across individuals. Individual differences in spatial abilities have been shown to be predictive of successful careers in science and technical disciplines (Shea et al. 2001; Wai et al. 2009) and associated with specific academically relevant skills, such as mathematical thinking (e.g., Frick 2019; Kyttälä and Lehto 2008; Laski et al. 2013; Reuhkala 2001; for a review see Mix and Cheng 2012). Although some possible mechanisms have been proposed that could explain these connections (e.g., Hawes and Ansari 2020), further research is needed to test these accounts. Yet, it remains difficult to find validated measures for many specific types of spatial abilities (cf. Brucato et al. 2022; Newcombe et al. 2023), especially if they need to be suitable for young children or group testing. Many of the existing measures that closely model tests originally developed for adults are difficult to understand for young children or preschoolers. Yet, for tests that have been adapted to be more child-friendly, it is still unclear whether they measure the same ability, because direct cross-validations with established measures that are typically used in research with adults are largely missing. Thus, study designs in developmental research are often limited by the fact that no validated age-appropriate assessment tools exist. To advance research on the development of spatial abilities and how individual and age-specific variations in spatial skills predict proficiency in other domains, we need age-appropriate assessment tools with acceptable psychometric properties (cf. Newcombe et al. 2023). 

Therefore, in the present study, five spatial tasks were presented to children between 6 and 10 years of age. Two of these were developed to measure mental rotation (MR) in children, one in the format of a computerized chronometric tasks and one in a paper–pencil format that is suitable for group assessment. To establish convergent validity, these two tasks were compared to two established MR assessments for adults in computerized and paper–pencil format, respectively. The MR tasks were also compared to a task measuring perspective taking (PT), in order to determine discriminant validity. In addition, children’s verbal skills, socioeconomic background, and video game use were assessed by means of a parent questionnaire, as previous research had suggested that these variables may be relevant for MR or PT performance. For example, Levine et al. (2005) found that boys from middle and high socioeconomic backgrounds outperformed girls on a MR task, whereas boys and girls from low socioeconomic backgrounds did not differ in their performance. Frick (2019) found that PT (but not MR) performance was positively related to children’s socioeconomic status and verbal IQ. Moreover, Quaiser-Pohl et al. (2006) found lower MR scores for boys who did not play video games as compared to action-and-simulation game players. To obtain a more complete picture of children’s media use, television consumption was also assessed in the present study.

### 1.2. Mental Rotation (MR)

Among the varieties of spatial skills, MR—the ability to imagine an object in an orientation different from the one perceived—has been of particular importance and highly indicative of performance in the mathematical domain. For example, a cross-sectional study with diverse samples revealed that MR predicted math aptitude, especially for female undergraduates and even when verbal skills were taken into account (Casey et al. 1995). Longitudinal data (Frick 2019) further showed that children’s MR skills in kindergarten and first grade were predictive for math performance (arithmetic operations) in second grade. Moreover, Cheng and Mix (2014) found that training MR improved math performance in 6- to 8-year-olds. 

In order to plot a continuous developmental trajectory of MR ability, it is necessary to have assessment tools that measure the same ability at different ages (cf. Frick et al. 2014a). However, a closer look at the MR tests employed in developmental research shows that measures vary considerably between studies and age groups (cf. Levine et al. 2016). Thus, the question arises of whether these tests measure the same ability and whether this is commensurate to MR ability assessed in research with adults. In a classic MR task (Shepard and Metzler 1971), adults were presented with two drawings of cube-shaped objects in different orientations (similar to those depicted in Figure 3). Their task was to decide whether the two objects were mirror images that could not be rotated into congruence (“different”) or identical objects that could be rotated into congruence (“same”), by pressing one of two buttons. This task inspired many MR tasks that were adapted for the use in children by presenting more child-friendly stimuli (e.g., Estes 1998; Jansen et al. 2013; Marmor 1975). However, these kinds of tasks have proven rather difficult for many children below the age of about 5 years (for an overview, see Pedrett et al. 2023). Presenting mirror images has the advantage that the two stimuli have the same features by design and, thus, cannot be differentiated using feature strategies. Yet, the concept of *mirror images—*or what constitutes *same* or *different* objects in an MR task—is difficult to explain to young children or preschoolers. 

Therefore, many MR tests used in developmental research have deviated from this principle. For instance, in a paradigm that has been adopted for developmental research (e.g., Gunderson et al. 2012; Ramirez et al. 2012) due to its ease of instruction, participants are asked to select the figure (out of four) that, when combined with a reference figure, will make a certain shape (e.g., a square in the MR subtest of the Primary Mental Abilities Test, Thurstone 1938; Thurstone and Thurstone 1949). Inversely, the Children’s Mental Transformation Task (Levine et al. 1999) requires children to choose which of four shapes would result from combining two figures. This task has later been used to assess MR in developmental research (e.g., Ehrlich et al. 2006). However, the choice alternatives can be differentiated based on spatial features (cf. Levine et al. 2016), allowing participants to solve the task without MR. Even some paper–pencil tasks that are widely used in MR research with adults do not (or not only) present mirror images (e.g., Vandenberg and Kuse 1978), opening the door for feature-based strategies (Hegarty 2018). Thus, results from non-mirror-image tests should be interpreted with caution.

Other paradigms used in developmental research (Frick et al. 2013a, 2013b) presented mirror images as stimuli but used a puzzle-like paradigm, to avoid having to explain the concept of mirror images and to make the task easier to understand for younger children. Instead, children were shown two two-dimensional mirror images and were asked to choose the one that would fit into a hole that was presented in a different orientation. Even though these paradigms cannot be solved via feature-based strategies, the question remains whether they measure the same ability as the original MR task. 

Therefore, the first purpose of the present study was to directly compare the *Ghost Rotation Task* (Frick et al. 2013b), which uses a puzzle-like paradigm, to a classic MR task. Establishing the validity of puzzle-like MR paradigms that use mirror images provides new avenues for developmental research, as such tasks can be solved above chance level by children as young as 3.5 to 4 years of age (cf. Pedrett et al. 2023). A second purpose was to develop and validate a paper–pencil version of the *Ghost Rotation Task* that is appropriate for a broad age range and suitable for group testing. Many developmental studies are conducted in school settings where one-on-one testing of individual children is typically very limited or often not possible. Having a child-friendly paper–pencil task that can be efficiently administered in groups could be very useful for school-based research. 

To cross-validate these newer tasks, two established assessments were used, that had been previously employed in similar form in research with adults. One was a computerized chronometric task based on the original task paradigm introduced by Shepard and Metzler (1971). We expected this task to correlate positively with a computerized chronometric version of the *Ghost Rotation Task*.

The *Figure Rotation* task was used as a second benchmark task for cross-validation in the present study. It is a timed paper–pencil task from a task battery that was famously used to assess cognitive development in older adults in the *Seattle Longitudinal Study* (*Schaie-Thurstone Adult Mental Abilities Test*, Schaie 1985). This task was deemed appropriate for children at this age, as it was developed for older adults, hence featuring larger stimuli with more space in between, rendering it less visually confusing as compared to other paper–pencil tasks (e.g., *Card Rotations Test*; Ekstrome et al. 1976). The *Figure Rotation* task has been used successfully with 8-year-olds in previous research (Frick 2019). Unlike other paper–pencil MR tasks (e.g., Vandenberg and Kuse 1978; Thurstone and Thurstone 1949) this task only presents mirror images and, thus, alternative feature-based strategies can be ruled out more easily. We expected the *Figure Rotation* task to correlate positively with the paper–pencil version of the *Ghost Rotation Tasks*.

### 1.3. Perspective Taking (PT)

Visual PT—the ability to mentally adopt a viewpoint different from one’s own— has been investigated extensively in developmental research, originating with Piaget and Inhelder’s (1956) foundational work on *The Child’s Conception of Space*. Conceptually, MR and PT are highly similar, in that they are both dynamic spatial skills that require a mental transformation resulting in an altered view of an object or configuration. Yet, the two abilities are thought to require slightly different kinds of transformations that rely on distinct cognitive processes. MR relies on object-based transformation and typically involves a mental change in the orientation (and sometimes position) of an object or array in the environment, while maintaining one’s spatial relation to this environment. PT on the other hand relies on viewer-based transformation and involves imagining a change of one’s own position (and often orientation) in the environment, while maintaining the spatial relations among everything else within this environment (cf. Hegarty and Waller 2004; Lohman 1979). The two processes have been found to be associated with distinct neural activation patterns (e.g., Committeri et al. 2004; Lambrey et al. 2012; Zacks and Michelon 2005), and behavioral research has shown that, even though MR and PT are correlated, they are still clearly dissociable skills (e.g., Hegarty and Waller 2004; Kozhevnikov and Hegarty 2001). Moreover, PT develops much more slowly in childhood (Frick et al. 2014b) and continues to improve in adolescence (Dumontheil et al. 2010). PT can therefore be considered a hard touchstone for establishing the discriminant validity of MR tasks, as it is a clearly dissociable yet very similar construct. We thus expected performance on the MR tasks to correlate to performance on a PT task, but these correlations were expected to be less strong than the ones among the different MR tasks.

## 2. Materials and Methods

### 2.1. Participants

In total, 108 children were tested. Data of 12 children were excluded from analysis because they had missing data on one or more of the spatial tasks due to language (7) or technical (1) problems, two children did not follow instructions, and two children were ill on the day of group testing. The remaining sample (*N*= 96) consisted of 21 6-year-olds (*M_age_* = 6.6 years, *SD* = 0.2, range: 73–83 months, 9 males), 24 7-year-olds (*M_age_* = 7.5 years, *SD* = 0.3, range: 86–95 months, 12 males), 24 8-year-olds (*M_age_* = 8.4 years, *SD* = 0.3, range: 96–107 months, 11 males), and 27 children between 9 and 10 years of age (*M_age_* = 9.5 years, *SD* = 0.4, range: 108–123 months, 12 males). That is, 4 children were already 10 years old but grouped together with the 9-year-olds; for simplicity, this combined group will be referred to as 9-year-olds in the following.

Children were recruited through elementary schools in or near a large city in Switzerland. They were tested in Swiss–German. Parents or legal guardians were informed about the study in a letter and provided written consent; children provided verbal assent. Children received a small gift or snack for their participation. The study followed ethical guidelines and was approved by the Institutional Review Board of the University of Fribourg. 

### 2.2. General Procedure

The children were tested at school. In a first session that lasted about 45 min, the children were tested individually in a separate room. The experimenter was present during the whole session, seated orthogonally to the child, and provided standardized verbal instructions. Tasks were introduced as “games”. Children were praised for their performance after each game regardless of how well they did. 

The computerized tasks were performed on a tablet computer (HP Pavilion x360 Convertible) with a touch-sensitive 15.6” display (39.6 cm, full HD, multitouch). The tablet computer was placed flat on a table in front of the child at a viewing distance of about 30–40 cm. Task were presented using the Octave software (GNU Octave 4.0.0; Eaton et al. 2015) with Psychtoolbox (Brainard 1997) on an Ubuntu (LINUX) operating system.

Task order was held constant across participants and sessions, which is common for individual-differences approaches (cf. Wilhelm et al. 2013). The individual tasks, which will be described in detail blow, were presented in the following order: Ghost Rotation Task (5–8 min), Multitasking (7–8 min), AID-3 Synonyms subtest (approx. 4 min), classic Shepard–Metzler MR task (5–8 min), Perspective-Taking Task for Children (6–10 min), and AID-3 Antonyms subtest (approx. 4 min). Children completed an additional task for piloting purposes, which will not be further detailed here.

Within one week after the individual session, a group assessment was conducted, in which the children solved two MR tasks: the Figure Rotation task followed by the paper-pencil version of the Ghost Rotation Task. During the group testing, the experimenter was constantly present and provided verbal instructions. Group sizes varied from 2 to 14 children due to practical reasons.

### 2.3. Administered Tasks

#### 2.3.1. Ghost Rotation Tasks: Computer Version (GhostRotPC)

MR was assessed using a computerized version of the Ghost Rotation Task (Frick 2019; Frick et al. 2013b), which allowed for assessing response accuracy as well as response times per trial. First, two instruction trials were presented on an A4-sized sheet of paper. On the first instruction trial, a key was shown in the upper half of the sheet. The lower half showed two “holes”, that is, black circles (7 cm in diameter; centers 11 cm apart) with cutouts in the shape of the outline of either the key or its mirror image. The child was asked to point to the hole the key would fit into. The child was then asked to verify the response by using a transparency that showed the outline of the key. This could be placed over the key and then slid over to the hole. In a second instruction trial, the key was shown at an angular discrepancy of 180° relative to the holes. Therefore, it had to be mentally rotated 180° in the picture plane to match one of the holes. The child was again invited to select the matching hole, and then to check the response by rotating the transparency from the key to the selected hole. 

**Figure 1:**
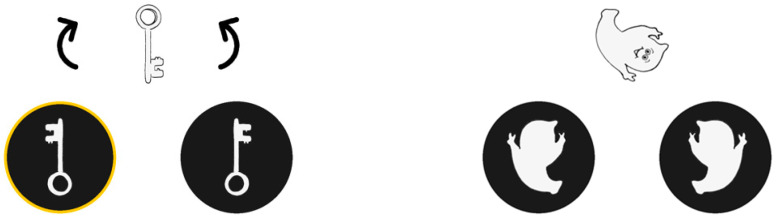
Examples of practice trials (**left**) and test trials (**right**) of the computerized Ghost Rotation Tasks.

Next, six practice trials were presented on the touchscreen computer. Children initiated each trial by pressing two fingerprints simultaneously with their two index fingers. The fingerprints were displayed 1.3 cm below each black circle and disappeared when pressed. Half a second later, an object (key, sock, ship, skate, hammer, or ghost) was shown in the upper half of the screen in various orientations, and two mirror-image holes were presented in the bottom half (see Figure 1, left). Children were instructed to first pretend rotating the top image in their head, and hence figure out in which hole it would fit, and only then to point to the hole. After selecting a hole, the choice was highlighted by a surrounding yellow circle, and the object could be rotated by pressing two arrows displayed on the touchscreen. 

The practice trials were followed by three test blocks with seven trials each, showing various ghosts in 7 different angular discrepancies to the hole (from 0° to 180°, in steps of 30°). The sizes of the ghosts varied between 5 and 6 cm at their longest extension. The 21 trials were presented in the same quasirandom order to all children. Arrow keys were no longer present (see Figure 1, right) and children received no feedback. Children were instructed to press the correct hole as quickly as possible but to also look very carefully and choose the correct hole. 

As a measure of performance, each child’s total number of correct responses was divided by the child’s accumulated response time across all trials. This measure thus reflected both the speed and the correctness of responses. 

#### 2.3.2. Ghost Rotation Task: Paper–Pencil Version (GhostRotPP)

This task was also adapted from the Ghost Rotation Task (Frick 2019; Frick et al. 2013b) but developed specifically for assessments in group settings. The paper–pencil task was presented with an overall time restriction but did not allow for analyses of response times on a trial basis. 

The test administrator first demonstrated the principle of the task in front of the class, using an A3 sheet of laminated cardboard which showed two rows of stimuli in the front and three rows in the back. The children had the same five rows on a A4 sheet of paper in front of them. Each row contained a reference figure (key, ship, hammer, sock, or skate) at the very left, and five target figures on the right, which were either the same as the reference figure or its mirror image. A vertical line divided the reference figure from the target figures. The children were asked to indicate for each figure on the right, whether it would match the reference figure, by circling the matching shapes and crossing out the nonmatching ones. To be able to distinguish nonresponses from errors, children were asked to mark both matching and nonmatching shapes. However, they were informed that their markings did not need to be beautiful (e.g., crooked lines and open circles were okay). The first two practice rows only contained upright figures; the last three rows showed the target figures in different orientations. For the first three practice rows, the test administrator visualized the task by laying an identical shape printed on a transparency over the reference figure and sliding it over the target figures, or sliding and rotating it in the picture plane in row three. The test administrator commented for each target figure whether it was a match or not, and circled it or crossed it out, respectively. Then, the children were asked to solve the last two rows with rotated target figures on their own, followed by feedback about the correct solutions, which the test administrator marked on her A3 cardboard.

**Figure 2:**
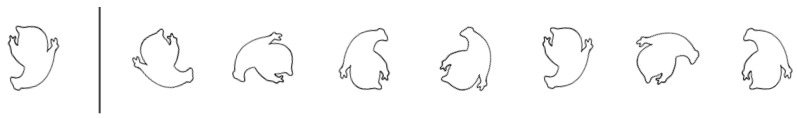
Exemplary test item of the Ghost Rotation paper–pencil task.

For the actual test, children were given two more A4 pages, each containing ten rows of ghost stimuli (see Figure 2 for an example row). Here, the reference ghost on the left had to be compared to seven ghosts on the right, which differed from the orientation of the upright reference figure by 0° to 180°, in steps of 30°. The shapes of the ghosts varied across rows. In total there were 20 different shapes. The children were allotted 2 min to solve each page. Half of the children solved the task in reverse page order, to discourage them from peeking at their neighbors’ sheet. A performance score was calculated by subtracting the number of incorrectly circled ghosts and incorrectly crossed-out ghosts from the number of correctly marked ghosts, summed across the two pages. Negative values were therefore possible, as for random responding a score near 0 could be expected. However, only one 6-year-old had a negative score (−2). The highest possible score was 140. Three children had only solved one of the two pages; therefore, regression-based imputation was utilized to estimate performance scores for these children. To that end, using the data of the remaining children, the (total) performance score was regressed on the performance score of the respective page, thus taking into account a possible difference in the difficulty of the two pages.

#### 2.3.3. Shepard and Metzler Cube Rotation Task: Computer Version (CubeRotPC)

A second computer-based MR task was presented, based on the original task by Shepard and Metzler (1971) but using 16 unique forms from a systematic library of redrawn cube stimuli by Peters and Battista (2008). The cube stimuli consisted of 10 to 13 cubes that were arranged to form four orthogonal “arms” (with three “elbows”). These 16 stimuli were then paired with either the same image or its mirror reflection in different orientations. 

To lower the cognitive demands for children, task instructions were held parallel to the computer version of the Ghost Rotation Task as much as possible. First, two instruction trials were presented on A4 paper sheets. Unlike the Ghost Rotation Task, where the children had to select the matching hole, this task required children to decide whether two figures presented in the top half of the screen were the same or different, that is, whether they would overlap completely or not match if turned in picture plane. If the figures matched, children were asked to press a blue response button with a tickmark; otherwise, they were asked to press a red button showing an x-mark. A yellow circle highlighted the selected response. Then, the correctness of the responses was checked by placing a transparency on the left figure and moving (or moving and rotating) it over the one on the right. 

Because this task format was somewhat more demanding as compared to the Ghost Rotation Task, 11 practice trials were presented on the touchscreen computer. The first 3 practice trials introduced the response buttons. Two upright (0°) cube stimuli were presented side-by-side and were either the same (first and last trial) or mirror images. The cube stimuli varied in size from 5 to 6 cm at their longest extensions and their center of rotation was 7 cm apart. The response buttons (3.9 cm in diameter, centers 16 cm apart) were presented in the lower half of the screen, 2 cm above the position of the fingerprints, and about 3 to 4 cm lower than the cube figures.

**Figure 3:**
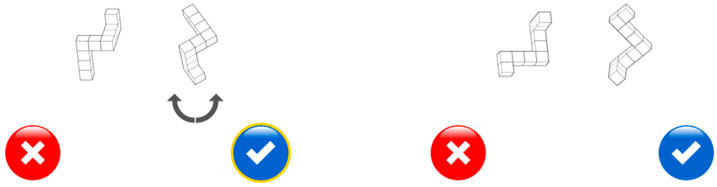
Examples of practice trials (**left**) and test trials (**right**) of the Shepard and Metzler cube rotation task.

After selecting a response button, children were invited to check the response by rotating the figure on the righthand side using the arrows (see Figure 3, left). Next, 6 practice trials (3 same) were presented with figures at angular discrepancies of 30°, 60°, 150°, 240°, 210°, and 300°. Practice trials were followed immediately by four test blocks that comprised 8 trials each (4 same), at angular discrepancies of 0° to 315°, in steps of 45° (see Figure 3, right). The 32 test trials were presented in quasirandom order per block. Each orientation was presented as a same trial in two blocks, and as a different trial in the other two. 

As a measure of performance, each child’s total number of correct responses was divided by the child’s accumulated response time across all trials, analogous to the approach in the computer version of the Ghost Rotation Tasks.

#### 2.3.4. Figure Rotation Task: Paper–Pencil Version (FigureRotPP)

To cross-validate the newly-developed paper–pencil version of the Ghost Rotation Task, the Figure Rotation subtest of the *Schaie–Thurstone Adult Mental Abilities Test* (STAMAT, Schaie 1985: test-retest reliability = .81 in adults) was used. The instructions were slightly adapted and supplemented with visual demonstrations, in order to make the task more accessible for this younger age group. The test consisted of rows of abstract 2D figures, and children were asked to decide whether each of six figures on the right was the same as or the mirror image of a standard figure presented on the left. Six practice rows from the original task manual were presented on a A3 laminated cardboard. The first three rows were explained by the test administrator using a transparency with the shape of the reference figure printed on it. The last three rows were also printed on A4 pages and children solved them alone, followed by feedback about the correct solution, which the test administrator marked on her A3 cardboard. Unlike the original task instructions that only required circling the matching figures, children were also asked to cross out the non-matching ones. This was done in order not to confuse the children with a different instruction than in the Ghost Rotation paper–pencil task and so that it was clear which items they had attempted to solve. 

For the actual test, two A4 pages with ten rows each were presented, and children were allotted 2 min to solve each page (instead of the 5 min allotted for the entire original test). Half of the children solved the task in reverse page order to discourage copying from their neighbors. A performance score was calculated by subtracting the number of incorrectly marked figures and incorrectly crossed out figures from the number of correctly marked ones. The maximum score was 120, whereas a score of 0 indicated random performance. Three children had negative scores (of −6, −4, and −2 at ages 7, 6, and 7, respectively). Six children had only solved one of the two pages. The same regression-based imputation method as in the paper–pencil version of the Ghost Rotation Task was used to estimate those children’s performance scores.

#### 2.3.5. Perspective-Taking Task for Children: Computer Version (PerspectivePC)

Spatial PT was assessed in the present study, using a computerized version of the *Perspective-Taking Task for Children* (PTT-C: Frick et al. 2014b). For the instruction, two Playmobil figurines, Lisa and Peter, were positioned on a sheet of white A4-sized cardboard. The two figurines each held a camera and took a picture of two geometric objects, a blue cylinder and a green cone, from an angle that differed by 90° and 180° from the child’s perspective. In four instruction trials, the children were shown an image of the objects from a particular perspective (0°, 90°, 180°, and 270°), and asked whether Lisa or Peter—or neither of them—could have taken that particular picture. To check the answer, the children were then asked to walk behind the figurines and peek over their shoulders. 

**Figure 4:**
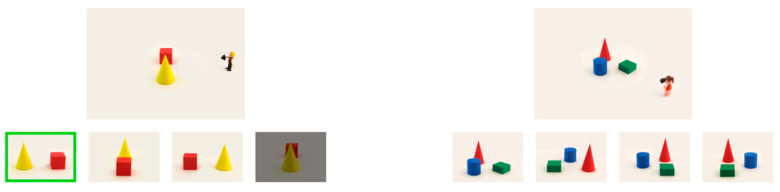
Examples of stimuli from the perspective -taking task: a practice trial, in which Peter is taking a picture of two objects from a 90° perspective (**left**), and a test trial from block 4, in which Lisa is taking a picture of three objects from a 45° perspective (**right**).

These three-dimensional practice trials were followed by three practice trials on the touchscreen computer. A scene (7.8 cm × 11.2 cm) was displayed in the upper half of the screen, featuring one figurine taking a picture of two geometric objects (red cube and yellow cylinder). The children were then asked to think about how a photo, taken by the figurine from that position, would look. A choice of four photos (3.5 cm × 5 cm) was presented in the lower half of the screen. As the photos were now on the computer and the children could not walk behind the figurine and peek over its shoulder, they were instructed to pretend doing this in their heads. If the children pressed the incorrect picture, it was dimmed; if they chose the correct picture, it was highlighted with a green frame (see Figure 4, left).

Next, 28 test trials (see Figure 4, right) were presented in four blocks of increasing difficulty. The first test block showed 6 trials with only one object (another figurine with distinct front, side, and back views), which was photographed from the perspectives of 0° (twice), 90°, 180° (twice), and 270°. The second test block showed 6 trials presenting two geometric objects, which were photographed from the same perspectives in a different order. The third test block showed 8 trials with three geometric objects, which were photographed from the perspectives of 0°, 90°, 180°, and 270° twice each. The fourth test block showed 8 trials with three geometric objects, which were photographed from the perspectives of 45°, 135°, 225°, and 315° twice each. All children saw the same quasirandom sequence of trials. During the test trials, the children did not receive any feedback. The ratio of correct responses relative to the total number of trials was used as the performance score.

#### 2.3.6. Verbal-IQ

Verbal-IQ was assessed using the subtests ‘finding synonyms’ and ‘finding antonyms’ from the AID-3 scale (Adaptives Intelligenz Diagnostikum 3, Kubinger and Holocher-Ertl 2014). In the ‘finding synonyms’ subtask, the children were asked to find a word that meant the same as a word read to them by the experimenter; in the ‘finding antonyms’ subtask they were asked to find a word that meant the opposite. There was one example and one practice item at the beginning. Children were presented with three blocks of five words. In this adaptive task, the starting block depended on children’s age, and response accuracy determined which two blocks followed. Following the test manual, raw scores were transformed into age-normalized T-scores and averaged across both subtasks.

#### 2.3.7. Media Use (TV and Games)

Children’s media use was assessed by means of a parent questionnaire. Parents were asked how much time (in minutes) the child spends watching television (TV) on a typical day of the week and on a typical day of the weekend (using TV sets, computers, or mobile devices). The same questions were asked with respect to video games (played on game consoles, computers, or mobile devices). The time provided for a weekday was multiplied by 5, the time for a day of the weekend was multiplied by 2, and then the two times were summed to obtain an estimate for the weekly usage. Parents provided information on TV usage for all but one child; no information on playing video games was obtained for 25 children. 

#### 2.3.8. Socioeconomic Status (SES) 

SES was determined based on parent’s occupations, coded using the ‘International Standard Classification of Occupation’ (ISCO-88, International Labour Office 1990) and converted to the ‘International Socio-Economic Index’ (ISEI; Ganzeboom et al. 1992). The ISCO and ISEI codes provide a fine-grained, continuous, and internationally comparable measure of SES. The higher index of both parents was used. If none of the parents reported an occupation, or the occupation could not be classified, the ISEI of the occupations for which they were trained was used. SES could be determined for all but two children. 

## 3. Results

### 3.1. Descriptive Statistics and Analyses of Differences between Age Groups and Sexes 

Table 1 gives an overview of the main test variables with descriptive statistics across age groups, for boys, and girls. Abbreviated variable names will be used in the following, as defined in the subheadings of Section 2.3. As expected, performance on CubeRotPC was lower than on GhostRotPC, which was originally designed for adults (pairwise *t*-tests for the total sample: *t*(95) = 21.89, *p* < .001; for single age groups: all *t*s > 7.94, all *p*s < .001). In fact, there were only two children in the entire sample who had a (minimally) higher score on CubeRotPC than on GhostRotPC. A similar pattern emerged for the paper–pencil tasks. Both in absolute terms (see Table 1; *t*(95) = 12.92, *p* < .001) and in percentage, average results were higher on GhostRotPP than on FigureRotPP. 

**Figure 5:**
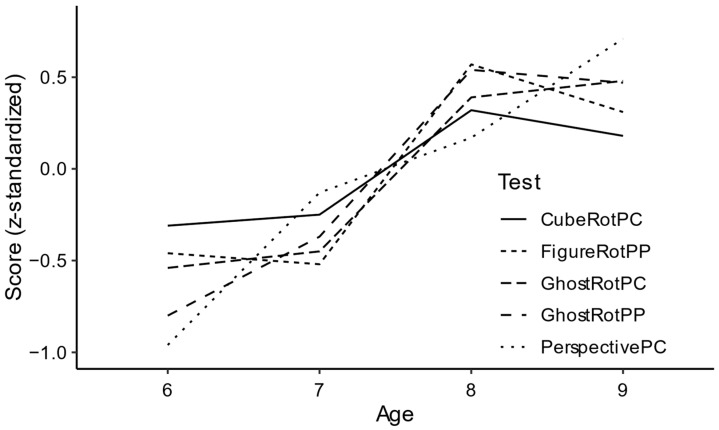
Age trends for the *z*-standardized performance measures of the spatial tasks.

In order to facilitate the analysis and comparison of age trends, participants’ scores on the five spatial tasks were z-standardized. As evident in Figure 5, performance on MR tasks increased sharply between 7 and 8 years of age but plateaued thereafter. In contrast, PerspectivePC performance improved almost linearly with increasing age, and still showed substantial progress between 8 and 9 years of age. These observations were tested for statistical significance by means of separate two-way analysis of variance (SS type II) for each task, with age and sex as independent variables. A square-root transformation was applied to PerspectivePC scores and CubeRotPC scores in order to meet the requirements of normally distributed residuals and variance homogeneity. Significant age effects were found for GhostRotPC, FigureRotPP, GhostRotPP, and PerspectivePC (all *F*s > 7.60, all *p*s <.001, all η_g_^2^ > .232) but only a nonsignificant trend for CubeRotPC, *F*(3, 88) = 2.40, *p* = .073, η_g_^2^ = .076. Post hoc Welch’s *t*-tests comparing consecutive age groups showed significant differences between 7- and 8-year-olds for all MR tasks (all *t* > 2.03, all *p*s < .002), including CubeRotPC, *t*(45) = 2.03, *p* = .048. No other age differences were significant for the five MR tasks (all *t*s < 1.55, all *p*s > .130). PerspectivePC performance, on the other hand, increased between 6 and 7 years of age, *t*(42) = 3.65, *p* < .001, as well as between 8 and 9 years, *t*(49) = 2.19, *p* = .033, but not between 7 and 8 years, *t*(43) = 1.40, *p* = .169. No significant sex differences were found for the spatial tasks (all *F*s < 1.17, all *p*s > .282, all η_g_^2^ < .013), except for GhostRotPC, which showed a significantly higher performance for boys than for girls, *F*(1, 88) = 7.61, *p* = .007, η_g_^2^ = .080. The ANOVAs revealed no significant interactions between sex and age (all *F*s < 1.74, all *p*s > .165, all η_g_^2^ < .056).

Separate age by sex ANOVAs were also calculated for the hours spent with TV and Games as dependent variables. A logarithmic transformation was applied to both variables in order to meet ANOVA requirements. Neither TV, *F*(3, 87) = 0.95, *p* = .420, η_g_^2^ = .032, nor Games, *F*(3, 63) = 0.95, *p* = .422, η_g_^2^ = .043, showed significant age differences. For Games, a significant effect of sex could be observed, *F*(1, 63) = 7.9, *p* = .016, η_g_^2^ = .088, with boys playing more hours per week than girls. No significant interactions of age and sex were found (all *F*s < 1.58, all *p*s > .202, all η_g_^2^ < .070).

Even after transformation, SES did not fulfill the ANOVA’s requirement of homogeneity of variances. Hence, only Walsh’s *t*-tests (which are relatively robust against unequal variances) were calculated for both main effects. Girls’ and boys’ parental SES did not differ, *t*(33) = −0.26, *p* = .792. No differences in parental SES between consecutive age groups were observed (all *t*s < .47, all *p*s > .642), with the exception of a significant difference between 8- and 9-years-olds, *t*(33) = 2.12, *p* = .042. 

For the Verbal-IQ T-Scores, no age differences were to be expected due to T-scores being age-normalized. Accordingly, the ANOVA revealed no significant effect of age, *F*(3, 88) = 1.62, *p* = .191, η_g_^2^ = .052. Moreover, there was no significant effect of sex, *F*(1, 88) = 0.70, *p* = .191, η_g_^2^ = .008, and no interaction, *F*(3, 88) = 0.48, *p* = .016, η_g_^2^ = .696.

### 3.2. Reliabilities

Cronbach’s alpha^1^ was calculated for all spatial tasks. Because the two paper–pencil tasks (FigureRotPP and GhostRotPP) were speed tests and many items remained unanswered (especially by younger participants), Cronbach’s alpha might be an inflated estimate of reliability in these cases. Therefore, split-half reliabilities were also calculated for the two paper–pencil tasks. To further minimize inflated reliabilities of paper–pencil tasks, we excluded the data of children who only solved one page. The results are presented in Table 2. 

Cronbach’s alpha varied from acceptable to excellent across all tasks and (sub-)samples, with CubeRotPC in age group 6 being the only exception. On average, Cronbach’s alpha of the paper–pencil tasks was higher than of computer-based tasks. Moreover, split-half reliabilities calculated for the paper–pencil tasks were lower than the corresponding Cronbach’s alpha values, and this discrepancy was larger for FigureRotPP than for GhostRotPP, suggesting that Cronbach’s alpha was indeed inflated. It is also noteworthy that both Ghost Rotation tasks were more reliable than the more established task, which were originally designed for adults. 

### 3.3. Correlations among Tasks

Pearson correlations and partial correlations controlled for age (in months) were calculated for each pair of variables. Again, logarithmic and square-root transformations were applied to Games, TV, PerspectivePC, and CubeRotPC values to achieve normally distributed data. Table 3 shows the Pearson correlations (below the diagonal) and partial correlations (controlled for age; above the diagonal) between all variables, as well as significance levels that were Bonferroni–Holm-corrected for multiple testing, respectively.

Among the four MR scores, correlation coefficients were all statistically significant with medium to large effect sizes, except for the correlation between CubeRotPC and GhostRotPP. Even though these coefficients were reduced in size after controlling for age, the general pattern remained the same. Correlations between the four MR scores and PerspectivePC were smaller in comparison, and when age was controlled for, none of the correlations remained significant. The correlation between PerspectivePC and the CubeRotPC was even close to zero.

A rather irregular pattern emerged for the (partial) correlations between spatial and nonspatial scores. None of the nonspatial variables were significantly related to CubeRotPC performance. TV consumption was, albeit not statistically significant, negatively associated with MR scores, with small but consistent negative relations. Games, on the other hand, showed a less consistent pattern.

Verbal-IQ scores showed weak- to medium-sized correlations with spatial abilities, with the exception of CubeRotPC. The paper–pencil tasks and PerspectivePC were also positively related to SES. However, these correlations were not statistically significant when age was controlled. When age-controlled, SES and Verbal-IQ were both negatively but not significantly related to TV consumption and (to a lesser extent) to Gaming.

### 3.4. Maximum-Likelihood Exploratory Factor Analyses

To obtain a fuller picture of the relations among the five spatial task scores, a factor analysis was conducted based on age-controlled partial correlations. The Kaiser criterion and Scree plot indicated only a single factor for a maximum-likelihood factor analysis, whereas parallel analysis suggested two factors. Hence, two separate analyses were conducted: one for a single-factor solution and one for a two-factor solution with oblique rotation. Factor loadings for both analyses are presented in Table 4. The single-factor solution accounted for 37% of the total variance (RMSEA = .054, χ^2^(5) = 6.43, *p* = .266). The two-factor solution accounted for 55% of the total variance (Factor 1: 34%, Factor 2: 21%; RMSEA < .01, χ^2^(1) = 0.28, *p* = .594). In both analyses, the first factor was primarily related to FigureRotPP, GhostRotPP, and GhostRotPC. CubeRotPC loaded moderately on the single factor. In the two-factor solution, on the other hand, CubeRotPC alone constituted the second factor. The two factors correlated with *r* = .32, which was nearly identical to the factor loading of CubeRotPC in the single-factor solution. GhostRotPC also showed weak cross-loadings on the second factor. Finally, PerspectivePC was always associated with the first factor, albeit only weakly.

Due to the above findings that some spatial scores were strongly related to verbal abilities, the two-factor analyses were repeated, including partial correlations with Verbal-IQ scores. The single-factor solution accounted for 32% of the total variance (RMSEA = .067, χ^2^(9) = 12.99, *p* = .163) and the two-factor solution for a little over 43% (Factor 1: 30%, Factor 2: 13%; RMSEA = .05, χ^2^(4) = 5.00, *p* = .288), with an interfactor correlation of *r* = .37. Factor loadings for both analyses are presented in Table 5. Most importantly, the pattern of factor loadings was nearly identical to the one obtained without Verbal-IQ. Again, the first factor was weakly associated with PerspectivePC and most strongly related to FigureRotPP, GhostRotPP, and GhostRotPC. CubeRotPC was moderately associated with the first factor (single-factor solution) or the main contributor to the second factor (two-factor solution). Again, GhostRotPC showed weak cross-loadings on the second factor. Verbal-IQ loaded moderately on the first factor in both solutions and was negatively related (but weakly) to the second factor in the two-factor solution. 

### 3.5. Response Times

In addition to the performance score used as an index of each individual child’s average performance, computer tasks also offer the possibility to analyze performance on trial-by-trial basis. As is typical for computerized MR tasks, response times were analyzed as a function of angle of presentation (i.e., the angular discrepancy between the presented stimulus and its upright reference figure). Response times for incorrect trials were included in order to have more data points (the same number as used for the above performance score), and average response times were highly similar with and without the incorrectly solved trials (Pearson’s *r* = .94, *p* < .001 for CubeRotPC, and *r* = .98, *p* < .001 for GhostRotPC). Mean response times by angle and age group are presented in Table 6 (GhostRotPC) and Table 7 (CubeRotPC). Overall, children responded faster on GhostRotPC than on CubeRotPC, *t*(95) = 12.84, *p* < .001.

An ANOVA with response times on GhostRotPC as dependent variable, the within-participant variable of angle (7), and the between-participant variable of age group (4) yielded significant main effects of angle, *F*(4, 358) = 23.43, *p* < .001, η_g_^2^ = .089, and age group, *F*(3, 92) = 3.35, *p* = .023, η_g_^2^ = .063, but no interaction, *F*(12, 358) = 0.88, *p* = .570, η_g_^2^ = .011.

An analogous ANOVA with response times on CubeRotPC as dependent variable, the within-participant variable of angle (5), and the between-participant variable of age group (4) yielded a significant effect of angle, *F*(3, 253) = 83.75, *p* < .001, η_g_^2^ = .294, but no effect of age group, *F*(3, 92) = 0.10, *p* = .958, η_g_^2^ = .002, and no interaction, *F*(8, 253) = 1.27, *p* = .259, η_g_^2^ = .019.

### 3.6. Accuracy

Below, the proportion of correct responses are reported, in order to facilitate comparison with other tasks in the literature. Table 8 and Table 9 show the proportions of correct responses by angle and age group for the two computerized MR tasks, Table 10 for the perspective-taking task. Again, there was a significant difference in overall accuracy between the GhostRotPC and the CubeRotPC, *t*(95) = 14.34, *p* < .001.

One-sided one-sample *t*-test comparisons against chance (.5) showed that children of all age groups performed above chance level on both MR tasks (all *t*s > 5.25, all *p*s < .001). Performance was also significantly different from chance (.25) on the perspective-taking task (6-year-olds: *t*(20) = .198, *p* = .036; all other age groups: *t*s > 5.73, *p*s < .001).

## 4. Discussion

The present study compared 6- to 9-year-olds’ performance on two mental rotation tasks that were specifically developed for children with their performance on two established tasks that were originally created for adults. Perspective-taking performance was also measured to determine discriminant validity with a different but conceptually related spatial task. In addition, children’s verbal skills, TV consumption, and gaming experience were assessed. 

### 4.1. Construct Validity

Correlational analyses showed significant positive correlations among all MR task. Correlation coefficients were moderate to strong, ranging between *r* = .37 and *r* = .68, if age was controlled, with the exception of CubeRotPC, which was correlated with coefficients between *r* = .21 and *r* = .43. Correlations between MR tasks and PT were substantially smaller and dropped below significance level when effects of age were partialled out. This pattern was confirmed by results of exploratory maximum likelihood factor analyses, showing that most MR tasks loaded highly on a common factor, whereas PT showed a substantially smaller factor loading. These findings suggest that convergent and discriminant validity of the new MR tasks was high, in that they measured the same ability as established MR tasks and a different ability than a closely related but conceptually different spatial task. 

One of the MR tasks (CubeRotPC), a computerized task which was originally designed to be used with adults, only showed moderate loadings on the common factor identified in the factor analysis. When a two-factor solution was forced, as suggested by parallel analysis, this task constituted a second factor on its own. However, the correlation between the two factors was high and about equal to the factor loading of this task on the single factor; hence, the two-factor solution did not yield any additional insight. The much lower performance scores (as compared to GhostRotPC) and the lower reliabilities suggested that this task was very hard for children, even in the oldest age group. One reason for this could be that this task presented three-dimensional cube figures, whereas stimuli in all other tasks were flat two-dimensional shapes (but note that all MR tasks required a rotation in the picture plane only). Another difficulty of this task is that the response had to be mapped onto a response button (with an abstract tickmark for matching and an x-mark for nonmatching stimuli), whereas on all other tasks, children could give unmediated responses, by pressing or marking the stimulus directly. The fact that the computerized Ghost Rotation Task also showed some cross-loadings on the second factor, may also suggest that this factor represented some aspect that arose from computerized presentation. 

### 4.2. Reliability

On average, the new MR tasks had good to excellent reliabilities and proved more reliable than the already established reference tasks for adults. For instance, the task that was based on the classic Shepard and Metzler (1971) task showed good reliability overall but dropped to nonacceptable (< .70) for the 6-year-olds, suggesting that it was too difficult at that age and children were mostly guessing. Future research might further investigate which characteristics are responsible for this difference in difficulty between the tasks. One explanation pertains to the abstract nature of the cubes and figures as compared to the ghost stimuli, which may have been more motivational for children. A second reason may be that the ghosts were easier to encode due to their closed shape (Gestalt). This also gave the ghost some bodily characteristics, which may have prompted a body-analogy that has previously been shown to facilitate mental rotation of objects (Amorim et al. 2006). 

On average, Cronbach’s alpha of the paper–pencil tasks was higher than of computer-based tasks. This was reduced when split-half reliability was calculated to account for nonattempted items on the speeded paper–pencil tasks. It is also noteworthy that reliability did not increase monotonically with age on most measures. This may be due to changes in strategies (e.g., speed/accuracy; holistic/piecemeal), which may be more common in critical developmental phases. The PT task showed excellent reliability for the total sample, and at least good reliabilities in all age groups. 

### 4.3. Developmental Aspects

Children’s’ performance on the new MR tasks was higher as compared to the respective tasks that were originally designed for adults. Significant age effects for GhostRotPC, GhostRotPP, and FigureRotPP suggested that these tasks were sensitive to demonstrate developmental changes in this age range, whereas CubeRotPC only showed a nonsignificant trend. Yet, all MR tasks showed significant performance gains between 7 and 8 years of age. In contrast, PT performance improved almost linearly with increasing age, with substantial developmental progression between 6 and 7 and between 8 and 9 years (but not between 7 and 8 years) of age. This asynchronicity in developmental progress further speaks to the discriminant validity of the new MR tasks. 

Computerized tasks have the advantage that response times can be assessed on a trial-by-trial basis, and effects of presentation angle on response times can be analyzed (cf. Estes 1998; Frick et al. 2009; Kosslyn et al. 1990; Marmor 1975). A significant linear increase in response times with increasing angular discrepancy between the two presented stimuli is typically interpreted as a sign of MR (e.g., Shepard and Metzler 1971). Increasing response times were found for both computerized tasks. Whereas response times on GhostRotPC decreased with age (especially between 7 and 8 years of age), there was no age effect on CubeRotPC, again suggesting that the latter task is not sensitive enough to register developmental changes in this age range. 

### 4.4. SES and Verbal-IQ

The two paper–pencil MR tasks and the PT task showed weak correlations to SES (albeit not significant in all cases, or if age was controlled). Similarly, performance on the two paper–pencil MR tasks and PT showed positive correlations to Verbal-IQ. This suggests that the computerized MR tasks were somewhat more culture-free than the other three spatial tasks. Encoding spatial relations in language (e.g., “the red square is on Peter’s right”; “the red cone is behind”) might have been especially helpful for PT performance. Moreover, although both ghost tasks presented the same stimuli, the paper–pencil format may have prompted a language strategy, as several of the same ghosts/figures (and several of their mirror images) were presented in one row. A language strategy may have a been particularly efficient way to encode and mentally represent the reference stimulus and compare it to *several* same or mirror-image versions. 

### 4.5. Watching TV and Video Gaming

More time spent watching TV was (not significantly but consistently) associated with lower SES and Verbal-IQ, as well as lower MR scores. Playing video games showed a less consistent pattern, although there was a significant positive correlation between TV and Games. One possible interpretation is that playing video games is a highly heterogeneous activity, as games differ in many aspects, including their spatiality. Thus, positive and negative effects may outweigh each other. However, it is important to bear in mind that media usage was assessed by means of parent questionnaires. Parents’ answers could have been biased by tendencies to respond in a socially desirable way (especially for children with a high level of media consumption), or their knowledge may have been inaccurate as (mobile) media consumption is difficult to monitor. Both factors could have reduced variance in these variables and, thus, also the size of the observed correlations to other variables. Future research might employ additional measures for media consumption (e.g., children’s self-reports) to further investigate the effects of media use on spatial performance.

### 4.6. Limitations

A limitation of the present study is that the sample cannot be considered representative or normative. For the main purpose of this study, to cross-validate the different mental-rotation and perspective-taking tasks, this is not problematic. However, it should be kept in mind that the scores provide only an estimate of typical performance levels in these age groups, and should therefore be used cautiously for diagnostic purposes.

A second limitation pertains to the paper–pencil tasks when used in group settings. With young children, it is possible that some children do not follow the instructions. For example, some children turned the sheets, so that the reference figure was on the right side. We, therefore, only had data from one part of the test for those (few) children. This happened less often in the Ghost Rotation Task, as in this task a vertical line separated the reference ghost from the other ghosts. In single settings or small groups, such and other irregularities can be better detected or prevented than in large group settings.

## 5. Conclusions

Overall, the new spatial tasks discriminated well between performance levels of different age groups and proved to be suitable and valid measures of MR and PT performance in the age range of 6 to 9 years, with good to excellent reliability. Thus, the new tasks investigated in this study can be useful for future research assessing MR in single and group settings (computer and paper–pencil versions of the Ghost Rotation Test, respectively) and PT in single settings (computer version of the Perspective-Taking Test for Children). Future studies might explore the age limits at which these tasks are no longer able to discriminate sufficiently between individual performance levels; however, since the scores suggested here account for response times, there may very well not be an upper limit.

## Figures and Tables

**Table 1:** Descriptive statistics (mean and standard deviation) of all assessed variables for the total sample, each age group, and for boys (m) and girls (f).

	Total		6		7		8		9		m		f	
	*M*	*SD*	*M*	*SD*	*M*	*SD*	*M*	*SD*	*M*	*SD*	*M*	*SD*	*M*	*SD*
**GhostRotPC**	0.50	0.13	0.43	0.13	0.44	0.11	0.55	0.12	0.56	0.12	0.53	0.12	0.47	0.14
**CubeRotPC**	0.23	0.07	0.21	0.06	0.22	0.06	0.25	0.07	0.24	0.06	0.24	0.07	0.23	0.07
**GhostRotPP**	62.29	24.83	42.48	23.95	53.02	21.52	75.79	21.04	73.95	17.44	63.34	24.66	61.41	25.17
**FigureRotPP**	34.49	16.41	26.92	15.29	25.94	16.04	43.88	12.72	39.65	14.53	36.13	16.02	33.11	16.77
**PerspectivePC**	0.53	0.22	0.31	0.14	0.50	0.21	0.57	0.17	0.69	0.19	0.54	0.23	0.52	0.22
TV	5.78	4.82	4.72	4.31	5.74	5.56	6.11	4.78	6.30	4.63	5.91	5.72	5.67	3.97
Games	4.98	8.68	3.32	6.55	3.58	5.48	3.96	5.25	8.20	13.05	7.92	11.34	2.58	4.53
SES	63.46	15.90	61.50	17.40	62.29	15.18	59.92	19.69	69.31	9.47	63.00	14.00	63.86	17.53
Verbal-IQ	53.98	8.25	50.95	9.62	54.20	7.71	53.85	7.68	56.25	7.75	54.78	6.61	53.31	9.43

*Note.* The maximally possible score of GhostRotPP is 140; that of FigureRotPP is 120.

**Table 2:** Cronbach’s alpha (and split-half reliabilities) of the spatial tasks for the total sample and per age group.

	Total	6	7	8	9
GhostRotPC	.86	.86	.71	.85	.83
CubeRotPC	.80	.67	.79	.86	.81
GhostRotPP	.97 (.91)	.98 (.94)	.97 (.88)	.97 (.91)	.95 (.82)
FigureRotPP	.95 (.79)	.94 (.70)	.93 (.64)	.94 (.84)	.96 (.79)
PerspectivePC	.90	.81	.88	.79	.88

*Note.* For GhostRotPP and FigureRotPP, split-half reliability is reported in brackets.

**Table 3:** Pearson correlations (below the diagonal) and partial correlations controlled for age (above the diagonal).

	1	2	3	4	5	6	7	8	9
**1 GhostRotPC**		.37 **	.44 ***	.48 ***	.12	−.21	−.09	.01	.17
**2 CubeRotPC**	.43 ***		**.21**	**.29**	**.02**	−.15	.02	−.04	−.01
**3 GhostRotPP**	.56 ***	**.30**		.68 ***	**.14**	−.25	−.16	.14	.27
**4 FigureRotPP**	.57 ***	.35 *	.74 ***		**.19**	−.17	−.11	.24	.28
**5 PerspectivePC**	.36 **	**.17**	.41 ***	.39 **		.03	−.09	.29	.28
6 TV	−.11	−.10	−.12	−.08	.13		.49 ***	−.26	−.22
7 Games	.04	.10	.00	.01	.10	.50 ***		−.16	−.15
8 SES	.08	.00	.21*	.29	.33 *	−.23	−.11		.40 **
9 Verbal-IQ	.26	.05	.35 *	.34 *	.36 **	−.17	−.10	.43 ***	

*Note*. *** *p* < .001, ** *p* < .01, * *p* < .05 (Bonferroni–Holm-corrected for multiple testing).

**Table 4:** (Rotated) factor loadings for the maximum-likelihood factor analyses based on the partial correlation matrix, with either one or two factors.

	Single-Factor Solution	Two-Factor Solution	
Score	*Factor 1*	*Factor 1*	*Factor 2*
GhostRotPC	.58	.50	.21
CubeRotPC	.34	.00	1.00
GhostRotPP	.77	.80	−.05
FigureRotPP	.87	.86	.01
PerspectivePC	.20	.22	−.05

**Table 5:** (Rotated) Factor loadings for the maximum-likelihood factor analyses with either one or two factors based on the partial correlation matrix including Verbal-IQ.

	Single-Factor Solution	Two-Factor Solution	
Score	*Factor 1*	*Factor 1*	*Factor 2*
GhostRotPC	.58	.47	.27
CubeRotPC	.33	.00	.83
GhostRotPP	.87	.84	.03
FigureRotPP	.78	.81	−.05
PerspectivePC	.22	.25	−.08
Verbal-IQ	.33	.41	−.17

**Table 6:** Mean response times (s) by angle and age group for GhostRotPC.

	Overall	0°	30°	60°	90°	120°	150°	180°
total	1.97	1.61	1.80	1.85	1.87	2.06	2.26	2.38
6	2.27	1.82	2.27	2.20	2.13	2.17	2.60	2.73
7	2.11	1.70	1.78	1.94	2.06	2.39	2.46	2.48
8	1.78	1.43	1.64	1.62	1.67	1.94	2.06	2.10
9	1.79	1.54	1.60	1.69	1.66	1.78	2.00	2.26

**Table 7:** Mean response times (s) by angle and age group for CubeRotPC.

	Overall	0°	45/315°	90/270°	135/225°	180°
total	3.25	2.03	2.59	3.51	3.96	3.83
6	3.20	2.23	2.66	3.23	4.01	3.57
7	3.30	1.98	2.84	3.70	3.83	3.65
8	3.18	1.86	2.49	3.48	3.83	4.00
9	3.30	2.06	2.39	3.60	4.16	4.05

*Note.* Response times for the equivalent angles in clockwise and counterclockwise direction are averaged.

**Table 8:** Mean proportion correct by angle and age group for GhostRotPC.

	Overall	0°	30°	60°	90°	120°	150°	180°
total	.91	.98	.99	.94	.91	.89	.84	.81
6	.88	.97	.98	.94	.86	.86	.73	.81
7	.86	.99	1.00	.88	.89	.86	.78	.65
8	.94	.99	1.00	.96	.93	.92	.94	.85
9	.95	.99	.98	1.00	.96	.93	.90	.93

**Table 9:** Mean proportion correct by angle and age group for CubeRotPC.

	Overall	0°	45/315°	90/270°	135/225°	180°
total	.71	.85	.84	.68	.59	.64
6	.63	.76	.77	.57	.52	.58
7	.67	.84	.78	.64	.54	.64
8	.76	.92	.91	.76	.60	.66
9	.77	.88	.90	.73	.69	.67

*Note.* Response times for the equivalent angles in clockwise and counterclockwise direction are averaged.

**Table 10:** Mean proportion correct by angle and age group for PerspectivePC.

	Overall	0°	45/315°	90/270°	135/225°	180°
total	.53	.93	.40	.47	.32	.44
6	.31	.88	.26	.12	.07	.18
7	.50	.89	.45	.40	.30	.41
8	.57	.94	.33	.56	.38	.49
9	.69	.98	.53	.71	.47	.61

*Note.* Response times for the equivalent angles in clockwise and counterclockwise direction are averaged.

## Data Availability

The datasets of the current study are available from the corresponding author upon reasonable request.

## Note

1 McDonald’s Omega was also calculated but could not be estimated for some items due to low variability (almost all children responded correctly). As these items differed across age groups, it was not feasible to remove them for the analysis. The Omegas that could be estimated were close to Cronbach’s Alpha; therefore, only Cronbach’s Alpha is reported.
